# Advancing pediatric healthcare in Brazil: establishing reliable reference intervals for serum immunoglobulins^⋆^

**DOI:** 10.1016/j.jped.2024.03.001

**Published:** 2024-03-10

**Authors:** Khosrow Adeli

**Affiliations:** Clinical Biochemistry, Pediatric Laboratory Medicine, The Hospital for Sick Children, University of Toronto, Toronto, Ontario, Canada

The determination of accurate reference intervals (RI) for serum immunoglobulins is indispensable for effective clinical decision-making in pediatric healthcare. These intervals provide vital benchmarks for assessing immune function and diagnosing immune-related disorders in children. However, the process of establishing reliable RI is complex and often constrained by methodological limitations, particularly in pediatric populations. Addressing this gap, a recent population-based study conducted in Cuiabá, MT, Brazil,^1^ aimed to define RI for serum immunoglobulins G, M, and E in Brazilian children aged 1 to 10 years. This editorial discusses the significance of this study and its implications for pediatric healthcare in Brazil while incorporating relevant citations from the scientific literature to support key points.

## Significance of reference intervals in pediatric healthcare

Reference intervals play a pivotal role in pediatric healthcare by providing clinicians with essential guidance for interpreting laboratory results and making informed clinical decisions. In the context of immunoglobulins, reference intervals enable clinicians to assess immune function, monitor disease progression, and guide therapeutic interventions in pediatric patients with immune-related disorders.^2^ Accurate reference intervals, tailored to specific age and sex groups, are essential for ensuring the validity and reliability of clinical assessments in children.^3^

Challenges in Establishing Reliable Reference Intervals: Despite their importance, establishing reliable reference intervals for serum immunoglobulins presents numerous challenges in pediatric populations. These challenges include limited sample sizes, demographic variability, methodological discrepancies, and the influence of external factors such as ethnicity and geographic location.^4^^,^^5^ Inadequate representation of diverse populations and insufficient adherence to standardized methodologies can compromise the accuracy and generalizability of reference intervals, undermining their utility in clinical practice.

The Canadian Laboratory Initiative on Pediatric Reference Intervals (CALIPER) represents a landmark effort in advancing pediatric healthcare globally. By leveraging a vast dataset comprising samples from ethnically diverse pediatric populations, the CALIPER project has contributed significantly to the establishment of comprehensive reference intervals for various analytes, including immunoglobulins, in children.^2^, ^3^, ^4^ However, while the CALIPER database offers valuable insights, its applicability to specific populations, such as Brazilian children, may be limited due to variations in demographics and environmental factors.^3^

The recent study conducted in Cuiabá, MT, represents a significant milestone in Brazilian pediatric healthcare. Utilizing a rigorous methodology endorsed by the Clinical Laboratory Standards Institute (CLSI), the study aimed to establish accurate and population-specific reference intervals for serum immunoglobulins G, M, and E in Brazilian children aged 1 to 10 years.^6^ By including a diverse sample population representative of the Brazilian demographic landscape, the study sought to address existing gaps in pediatric reference intervals and enhance the quality of clinical care for Brazilian children.

Implications for Clinical Practice and Future Research: The establishment of robust reference intervals for serum immunoglobulins holds profound implications for clinical practice in Brazil. Clinicians can now rely on accurate and population-specific reference values to interpret laboratory results, diagnose immune-related disorders, and guide therapeutic interventions with confidence.^6^ Moreover, the availability of comprehensive reference intervals facilitates the standardization of clinical assessments and promotes consistency in pediatric healthcare delivery across different healthcare settings.

Moving forward, further research is warranted to expand our understanding of immunoglobulin dynamics in Brazilian children and optimize pediatric healthcare outcomes nationwide. Longitudinal studies tracking immunoglobulin levels across different age groups, geographical regions, and ethnicities can provide valuable insights into developmental trends, environmental influences, and disease susceptibility.^2^ Collaborative efforts involving multiple research institutions and healthcare stakeholders are essential to ensure the continued advancement of pediatric healthcare in Brazil and address the evolving needs of the pediatric population.

In conclusion, the establishment of reliable reference intervals for serum immunoglobulins in Brazilian children represents a significant advancement in pediatric healthcare. By adhering to a rigorous methodology and incorporating diverse sample populations, the study conducted in Cuiabá has provided clinicians with invaluable tools for assessing immune function and optimizing clinical decision-making in pediatric patients. As Brazil continues to strive for excellence in pediatric healthcare, continued research efforts and collaborative initiatives will be essential to further enhance our understanding of immunoglobulin dynamics and improve healthcare outcomes for children across the country.

## Conflicts of interest

The author declares no conflicts of interest.
